# Challenges of international oncology trial collaboration—a call to action

**DOI:** 10.1038/s41416-019-0532-4

**Published:** 2019-08-05

**Authors:** Monica Tang, Heikki Joensuu, Robert J. Simes, Timothy J. Price, Sonia Yip, Wendy Hague, Katrin M. Sjoquist, John Zalcberg

**Affiliations:** 1 NHMRC Clinical Trials Centre, University of Sydney, Sydney, NSW Australia; 2 Department of Oncology, Helsinki University Hospital and University of Helsinki, Helsinki, Finland; 3 The Queen Elizabeth Hospital and University of Adelaide, Adelaide, SA Australia; 4 Alfred Health and the School of Public Health and Preventive Medicine, Monash University, Melbourne, VIC Australia

**Keywords:** Cancer, Drug development, Research management

## Abstract

International collaboration in oncology trials has the potential to enhance clinical trial activity by expediting the recruitment of large patient populations, testing treatments in diverse populations and facilitating the study of rare tumours or specific molecular subtypes. However, a number of challenges continue to hinder the efficient and productive conduct of both commercial and non-commercial international clinical trials. These challenges include complex and burdensome regulatory requirements, the high cost of conducting trials, and logistical challenges associated with ethics review, drug supply and biospecimen collection and management. We propose solutions to promote oncology trial collaboration, such as regulatory reform, harmonisation of trial initiation and management processes and greater recognition and funding of academic (non-commercial) clinical trials. It is only through coordinated effort and leadership from researchers, regulators and those responsible for health systems that the full potential of international trial collaboration can be realised.

## An introduction to international collaboration in trials

While the earliest modern clinical trials were conducted by single centres and local research groups, recent decades have seen significant growth in international collaboration in trials in oncology and other fields. This has been aided in part by the development of cooperative trials groups, which play a central role in leading international investigator-initiated (non-commercial) collaborative trials (Box 1).

The increase in international collaboration, facilitated by technological improvements in communication, data capture and storage through advances in email, social media and web-based clinical data collection systems for example, has been fuelled by an increased recognition of the advantages of international collaborative trials over traditional single or several-centre local studies. Enrolling patients across multiple regions expedites the recruitment of large patient populations to late-phase trials to generate scientific and clinical data in a timely manner, with the potential to prove or disprove the value of novel interventions or alternative treatment options for cancer treatment more rapidly. Conducting clinical trials in geographically diverse patient populations also aims to improve the generalisability of results across broader genomic, biological, ethnic and socio-cultural backgrounds. Involving countries with less trial experience in international collaborations has the potential to benefit less experienced hospital sites by providing the local capacity to conduct clinical research and offering patients access to novel treatments. Furthermore, working in international research groups promotes the exchange of the most recent medical information and adoption of the best clinical practices, facilitating learning and identification of new globally important research questions.

Collaborative international trials play vital roles in meeting specific regulatory or scientific areas of need in oncology, such as the demand for data to support therapeutic registration applications, validation of efficacy of treatments currently used in practice (but not registered for such) and/or novel combinations of already approved treatments, as well as the development of treatments for uncommon cancers and specific molecular subtypes. Many national regulators require local study data to demonstrate that novel agents are safe and efficacious in particular countries and ethnicities prior to approving treatments, which has led to the design of multinational clinical trials that are conducted collaboratively but that also generate individual country data to support licensing applications across multiple jurisdictions. International trials provide an opportunity to detect any regional differences in the effectiveness of therapies, especially if potential geographical differences are identified prospectively in the study design.^1^ If regional differences do exist, international trials conducted across multiple regions are better placed to detect these differences than separately conducted smaller trials in each region.^2^

International trial collaboration also allows research into rare tumour types that might not be diagnosed in sufficient numbers in a single region for adequate trial recruitment. Furthermore, many trials of biological agents target recruitment of enriched populations characterised by specific, selected molecular profiles rather than primary tumour sites. As each specific molecular subtype might only account for a minority of cancers, trials involving such super-selection of patients screened from a much larger pool can only be accomplished with multicentre trials that recruit patients across several countries.

Box 1. Cooperative trials groups in oncology

- Cooperative trials groups play a central role in leading international non-commercial collaborative trials. These groups are responsible for coordinating multicentre trials within their respective regions and also collaborate to conduct international trials.
- Collaborations between cooperative groups provide the framework and resources to conduct international investigator-initiated, academic trials to address clinically important questions that are not necessarily of interest to commercial entities.
- The Intergroup Model of collaboration delegates roles and responsibilities for each region to the respective participating cooperative group, with the aim of facilitating cost-effective and efficient trial activation and accrual.^40^
- Examples of regional and national cooperative trial groups in gastrointestinal cancer include:
  - The European Organisation for Research and Treatment of Cancer Gastrointestinal Tract Cancer Group (EORTC GITCG)
  - The Canadian Clinical Trials Group (CCTG) Gastrointestinal Disease Site Committee
  - The Australasian Gastrointestinal Trials Group (AGITG)

## Challenges of international trial collaboration

Despite the benefits of international collaboration in oncology and other areas of medicine, however, a number of challenges, such as regulatory requirements, high costs and logistical problems, continue to hinder the efficient and productive conduct of international clinical trials. Many of these challenges are not unique to the field of oncology and have been recognised in cardiovascular, neurological and surgical research, among others.^3–6^ Similarly, many of the issues are common to international commercial trials; however, they pose serious impediments for academic researchers, who do not have access to the same resources and administrative support mechanisms as commercial entities.^6^ These challenges will form the focus of this review, although many of them apply equally to commercially-led trials and to other areas of clinical research.

### Regulatory requirements

Regulatory requirements for clinical trials aim to promote the safe and appropriate conduct of clinical trials, to safeguard the wellbeing of participants and to provide assurance of research standards and data quality. Harmonisation of regulatory requirements to facilitate international collaboration in clinical trials also has the potential to reduce “drug lag”, the situation in which regulatory approval for new agents in certain countries lags behind that of the European Medicines Agency and the US Food and Drug Administration.^7^ However, the complexity of trial-related regulatory obligations, as well as their variability between different countries and regions, increases the burden of documentation and compliance on investigators. For instance, the European Union (EU) Clinical Trials Directive 2001/20/EC of April 2001 required that all clinical trials in the EU be conducted in compliance with the International Conference on Harmonisation Good Clinical Practice (ICH GCP) guidelines.^8^ Although the intentions behind such developments are laudable, there is evidence that incorporating these guidelines into regulatory requirements hampers the development and initiation of academic clinical trials. Obstacles posed by the EU Clinical Trials Directive include the requirement for single sponsorship of multicentre trials and increased auditing by regulators. In the years following the introduction of this directive, the number of new trials initiated by the European Organisation for Research and Treatment of Cancer (EORTC) fell, from 38 in 2001, to 19 in 2004, to 7 in 2005.^9^ In the UK, the number of clinical drug trial regulatory applications fell by 5% on average annually, predominantly driven by a sharp decrease in academic trial activities, while clinical trial application rates increased 6–8% per annum in North America and Asia.^10^ In response to these effects on clinical trial development and initiation, particularly of investigator-led trials, the EU Clinical Trials Directive has subsequently been modified, but whether these updates will reverse these trends is unclear.^11^ There are similar concerns that requirements mandated by the US Food and Drug Administration are not suitable for adoption in low-income countries that do not have the resources to comply with stringent regulations.^12^ Researchers are also concerned that the General Data Protection Regulation (GDPR), which was recently introduced in Europe to strengthen individuals’ digital privacy rights and harmonise national data protection laws of EU members, may preclude sharing of clinical trial data between countries inside and outside the EU and further impede clinical research.^13,14^

Complexities and disparities in regulatory processes between countries can affect the time taken to activate international trials, with potential ramifications concerning the timeliness of study results. An analysis of regional timelines to set up a global phase 3 breast cancer trial conducted in 44 countries reported that time to regulatory authority approval varied significantly between regions, ranging from a median of 26 days in North America to 236 days in South America.^15^ A comparison of clinical research regulations in four high-income countries with a strong tradition of clinical research (Finland, England, Canada and USA) identified common basic structures with significant variations in details and scope, resulting overall in a highly complex research regulation system.^16^ Given that all regulatory systems are underpinned by the same principles of protecting patient safety and assuring research standards, the country-specific differences probably do not correspond to genuine disparities in research regulatory needs, but more likely reflect local variations in the development of regulatory structures.^16^ However, instead of facilitating clinical research to improve patient care, such inconsistencies and the challenges they pose to cross-country collaboration might confer the opposite effect: that is, they hinder useful clinical research. By contrast, the regulatory burden for the provision of standard care is almost trivial despite the fact that quality of care for patients enrolled in clinical trials, as well as outcomes for patients managed at research-active institutions, is substantially improved in comparison.^17–19^

The challenge of meeting complex regulatory burdens is not experienced equally by all parties. Academic and industry sponsors have different capacities to address the regulatory as well as financial and operational obligations of international clinical trials, such as on-site monitoring (including site preparation of source documents for monitoring and audit review), international safety reconciliation and reporting. However, regulatory guidelines do not differentiate between clinical trials conducted for different purposes by different types of sponsor. For instance, the same regulatory standards are applied equally to industry-sponsored registration trials for novel agents and to academia-sponsored pragmatic trials aimed at improving existing patient treatments (comparative-effectiveness research), often with the unwanted effect of stifling the development of the latter. Indeed, it could well be argued that such pragmatic trials are less risky and associated with better outcomes than standard care. For instance, in a trial investigating a complex clinical scenario such as neoadjuvant treatment of localised gastric cancer (TOPGEAR), the control arm comprises “state of the art” evidence-based treatment reached through multidisciplinary consensus, which is likely to be more rigorous than standard clinical care provided to many non-trial patients.^20^

The scope of academic clinical research reaches far beyond gaining approval of an anti-cancer agent. Introducing a new agent to the market should not mark the end of clinical and translational research but should lead to further research. For example, research into optimal drug dosing and duration of administration, research on side effects and their management, drug interactions, tumour and normal tissue biological effects and cost-effectiveness, might be greatly important to patients and societies and to the further development of drugs. Trials exploring such parameters also face regulatory burdens, even when the safety risk to patients is probably not greater than that in the standard care.

### Financial support

With greater regulatory obligations come increased logistical demands and requirements for funding. Well-established non-industry funding sources are available in many high-income countries in the form of government agencies (e.g. the National Cancer Institute in the USA, National Cancer Research Network and Medical Research Council in the UK, National Health and Medical Research Council and Cancer Australia in Australia) and charitable organisations. These funding sources, however, are limited in developing countries, which are more reliant on industry funding to run clinical trials. The high cost associated with conducting international trials limits the abilities of investigators to initiate large-scale academic clinical trials using public funding sources. This is illustrated by a 10-fold difference in the proportion of international trials funded by academic and commercial sponsors: only 3% of non-industry-sponsored trials are conducted internationally, compared with 30% of industry trials.^21^ Academic sponsors are more likely to focus their energies on local trials, with non-industry-sponsored trials accounting for the majority (77%) of single-country trials, while industry-sponsored trials dominate international trials (80%).^21^ However, given the invaluable role of investigator-led academic trials in advancing cancer care, a reduced capacity to conduct international non-industry-sponsored trials has serious implications for the future direction of cancer research. Lack of funding has been reported as the most important barrier to academic clinical cancer research amongst oncologists in both high and low-middle income countries.^22^

### Insurance requirements

A common regulatory requirement is indemnity or clinical trial insurance coverage for non-negligent harm resulting from clinical research.^8^ The availability of insurance varies by country, and insurance requirements even for comparative-effectiveness trials (in which both arms of the study represent currently acceptable treatment) often impose a further financial burden on academic clinical trial sponsors, which can preclude involvement by centres without access to appropriate insurance.

### Contractual issues

The complex regulatory requirements for the conduct of international clinical trials impose a high workload on the coordinating centres and participating sites. The roles and responsibilities of trial-related activities need to be delineated with a great degree of detail. This need for complex contractual agreements to ensure clear delineation of responsibilities further increases financial and setup time requirements. Furthermore, for trials with both academic and industry collaboration, there might be different drivers between academic investigators and industry representatives. The involvement of more stakeholders results in more complexity when drawing up a mutually satisfactory contractual agreement in such areas as ownership of the data and any consequential intellectual property.

### Administrative challenges

An unavoidable administrative challenge that contributes to the lead time required to initiate international trials across linguistically diverse regions is the language translation of patient information consent forms and/or protocols. This process may be further prolonged if local institutional research boards or ethics committees require forward and back translation of trial documents. In addition, time to trial initiation may be lengthened if protocols require inclusion of cooperative-group-specific protocol appendices to satisfy local regulatory or clinical standards.

### Drug supply challenges

The logistical challenges of conducting international clinical trials are not limited to administrative activities, but also encompass practical concerns such as drug supply and distribution. Differing drug approval processes and healthcare systems in different countries can affect the ease of supply and access to treatments in different regions, due to different importation and related requirements. Variations in labelling, transport and storage requirements across different regions may contribute to longer timelines and increased costs for study activation, which can have a significant impact on the cost and feasibility of conducting clinical trials in different countries. In addition, in some jurisdictions, all drugs in both the control and experimental arms of a study, even when approved as ‘standard of care’ for the same disease, need to be provided for study protocols. For example, if a standard of care drug is reimbursed in one jurisdiction and not another, the cost of supplying the drug in the latter case will make the trial impossible in the absence of substantial funding.

### Biospecimen collection and research

In the current, evolving era of personalised medicine, a greater emphasis is being placed on collecting biospecimens for translational research to better understand prognostic and predictive markers of clinical benefit and treatment toxicities. There is ongoing tension between retention of tumour tissue for clinical and legal requirements versus shared use in exploratory research, not to mention the increasing demands for access to the material to test for trial eligibility. While there are efficiencies in centralised testing and research, movement of biospecimens across national boundaries, varying approaches to ethical issues and observation of cultural considerations, and disparities in regulatory requirements all increase the complexity of biospecimen use in the international trial effort. Sometimes, parallel biobanks or laboratories need to be setup in each region, which then necessitates quality assurance processes to ensure reliability and consistency in specimen storage and analysis procedures. Regardless, in international trials, standardised biospecimen processing, storage methods and analysis protocols are critical to minimise pre-analytical variables that would otherwise confound results. Sponsors are therefore faced not only with escalating logistics and contractual activities with collaborators and service providers for translational research, but also the management of biospecimens and their future use in research, at times extending well beyond the closure of a trial. Furthermore, sponsors must have ethically defensible strategies for the return of clinically relevant (and actionable) translational research results to patients or their families, which is complicated not only by geography and cultural issues, but also by the ability to contact surviving patients or their next-of-kin at the appropriate time.

### Data sharing

Cooperation between collaborative groups is invaluable in conducting global, large-scale clinical trials. Prospectively planned meta-analyses of multicentre trials using pooled data, such as the International Duration Evaluation of Adjuvant Chemotherapy (IDEA) collaboration investigating the duration of chemotherapy in stage III colon cancer, provide the capacity to answer clinical questions requiring large participant numbers.^23^ However, the time and resource-intensive task of harmonising data captured in different formats from various trials poses a challenge to meta-analyses using individual participant data (IPD). Prospectively designed IPD meta-analyses are well-placed to overcome this obstacle by pre-specifying formats and definitions for data collection.^24^ Despite traditional reluctance for researchers to share data, it is important to further promote systematic data-sharing practices, as this approach could prevent unnecessary duplication of trials and maximise the potential of existing clinical data.^25–27^ Web-based technology such as that developed by the Project Data Sphere initiative has already been utilised to facilitate data sharing and generate analytic platforms to allow the research community to share, integrate and analyse existing patient-level trial data.^28^ In addition, enhanced efforts to collaboratively collect and analyse pharmacogenomic data will help to interpret trial data from different regions and determine the generalisability of trial results to different populations.

### Ethical considerations

Access to oncology clinical trials, as with many other facets of healthcare, is not uniform across the globe. Currently, the median number of trials per capita conducted in high-income countries is more than 100 times the number in low-income countries.^21^ Furthermore, there are disproportionately more randomised clinical trials conducted for conditions with high disease burden in high-income regions compared with conditions burdening low-income regions.^29^ Cross-regional collaboration provides benefits to emerging sites—those countries with less trial experience—not only by providing access to novel medications, but also by providing an opportunity to work with experienced clinical trial organisations and potentially improving healthcare through investment in local infrastructure and training of investigators and healthcare personnel.^30^

However, the increasing involvement of less experienced countries raises ethical issues, particularly in the early stages of international trial participation.^31^ The infrastructure for ethical and regulatory oversight of clinical research is usually less well-established in developing countries, which might affect the ability of these countries to conduct ethically rigorous and high-quality research. In a survey of 670 researchers from developing countries, 25% reported that their studies did not undergo any review by an institutional review board, ethics board or Ministry/Department of Health in the country where the research was conducted.^32^ The lack of competent local ethics committee procedures has been identified as an important barrier to participation in academic research by oncologists in low-middle income countries.^22^

Disparities in education and economic development might complicate the process of informed consent in low-income countries due to limitations in health literacy and undue influence from financial incentives if compensation is offered for research participation. Additionally, international collaborative trials of cancers that are common in developed countries might be expanded to enrol patients in developing countries for commercial reasons, such as relatively lower operational costs and rapid growth of market size in developing countries.^33^ This scenario raises ethical concerns if trial participants from developing countries are unlikely to have access to the corresponding drugs on completion of the trial, as it is important for clinical research to be relevant to the health needs of the communities in which the research is conducted, allowing translation of evidence into standards of care.^34^ Furthermore, if the healthcare systems and supportive care standards differ significantly between the countries in which the trials are conducted and those in which the treatment is likely to be utilised, the generalisability of trial outcomes might be limited. Therefore, greater priority should be given to multinational trials that encompass relevant regions where the trial results are likely to be applied.

Although access to a global patient population represents a rich resource for oncology trials, trials across countries with significant income disparities should be conducted in an ethically and scientifically sound manner, despite significant challenges in resource availability and in assuring consistent and appropriate regulatory oversight.

## Recommendations

There is no doubt that the future of many oncology clinical trials, if not the future of clinical cancer research, lies with global collaboration. Such collaboration is essential to ensure that trials are conducted in a timely manner and to provide equitable clinical trial access to patients, irrespective of location. However, obstacles remain that hinder the conduct of efficient, reliable and fair international trials. These challenges need to be addressed through concerted efforts from various stakeholder groups, including those in academia, industry and government as well as regulatory agencies. Existing projects, such as Cancer Breakthroughs 2020 (formerly known as Cancer Moonshot 2020), have demonstrated the potential of cooperation between these groups to accelerate cancer research (Box 2).

### Initiatives for improving clinical trials

The growing complexity of clinical trials, driven by scientific, commercial and regulatory factors, such as the need to collect biospecimens to develop companion diagnostics for patient selection and stringent monitoring requirements, contributes to rising trial costs and reduced efficiency resulting from poor participant accrual.^35^ A number of initiatives, such as the Clinical Trials Transformation Initiative (CTTI) and the ‘Sensible Guidelines’ group, aim to simplify trial design and to address difficulties affecting current clinical trials in general, including international oncology trials.^36,37^ The CTTI aims to improve efficiency and quality in clinical trials by providing evidence-based, consensus-driven recommendations and resources to promote efficient, patient-centric clinical trials.^36^ Examples of CTTI projects include the promotion of central institutional review boards for multicentre clinical trials, guidelines to optimise the conduct of data monitoring committees and tools to improve the quality and efficiency of safety reporting.^38^ Some of the CTTI recommendations are echoed by the policy report of the Working Group to Facilitate International Cooperation in Non-Commercial Clinical Trials convened by the Organisation for Economic Cooperation and Development (OECD) Global Science Forum.^6^ Broader acceptance and adoption of evidence-based recommendations for clinical trial design and conduct such as the increased use of single, potentially specialised ethics committees or simplified risk-adjusted reporting of adverse events would facilitate more efficient collaboration in international oncology trials.

### Regulatory reform

Key obstacles to increasing cooperation between regional collaborative groups include variation in the regulatory requirements in different regions and the burden of meeting them, especially for sponsors of academic trials. The fundamental aims of regulatory requirements—to protect patients and ensure high research standards—must not be compromised, but the implementation of processes to achieve this end should reflect type of study and level of risk and must be balanced against onerous investigator responsibilities that might hamper the initiation and conduct of trials. A ‘one-size-fits-all’ approach to trial coordination and monitoring often leads to cumbersome trials with stringent administrative requirements, which local research teams in developing countries might not have the experience or capacity to address.^39^ On the other hand, differing guidelines and regulations for safety reporting and pharmacovigilance, both between countries and also between industry and non-industry bodies within countries, can result in double-reporting for sites and increase the burden and costs for investigators and cooperative groups with minimal if any benefits for patients or the community.^40^ Regulatory reform must entail simplification as well as harmonisation, as harmonisation alone will be counterproductive if it results in merging different regulatory requirements in a more complex combined document, rather than removing unnecessary elements. Rather than isolating the processes and regulatory requirements of clinical research from those of medical practice, it would be helpful to more closely align research regulatory guidelines to ethical requirements for standard clinical care. Hence, simplification and harmonisation of regulations have the potential to reduce unnecessary work and costs for investigators and medical institutes, which will foster greater participation in trials.

There is an urgent need to investigate the evidence behind regulatory requirements, and the impact of these requirements on clinical trial standards, with a focus on preserving and developing methods of quality assurance that are effective and proportionate to the risks and complexity of the trials.^34,37,41^ For instance, in response to concerns about labour-intensive and potentially low-yield source data verification practices mandated for all trials in the original version of the ICH GCP guidelines, a risk-based approach to quality management has been incorporated into the updated Integrated Addendum to ICH GCP, with the aim of encouraging more efficient approaches to clinical trial design, conduct and oversight.^42,43^ The updated EU Clinical Trials Regulation (CTR) aims to streamline clinical trial initiation and conduct in Europe.^11^ Initiatives in this updated CTR include the creation of a single European clinical trial registration portal, introduction of ‘one-time’ consent for use of patients’ data, tissues and biological samples, and inclusion of a new category of ‘low-intervention clinical trials’ with simplified, risk-proportional monitoring.^44^ The impact of the modification of these guidelines and regulations on clinical trial development and conduct remains to be seen as the initiatives have only recently been established.

### Other areas for harmonisation

Administrative aspects of clinical trial conduct can be optimised to support international collaboration. Significant progress has already been made to facilitate collaboration in oncology at the clinical level through the harmonisation of staging, classification, toxicity and end-point definitions.^45–49^ Further improvement could be achieved through greater use of centralised institutional review boards and harmonisation of guidelines with respect to data protection directives and privacy laws, record retention regulations, and biospecimen acquisition, ownership and storage.^31,50^ Efforts have been made to standardise contractual language for research agreements through the creation of ‘common language’ contract templates to shorten the contract negotiation time prior to opening clinical research trials.^51^ There is value in analysing different models of clinical trial oversight and conduct between different countries and regions, with the aim of adopting the most pragmatic, efficient and reliable aspects in a harmonised model.^16^

### Funding

As with all aspects of healthcare, the difficulty in funding clinical trial research exemplifies the enduring challenge of maximising the utility of finite health funding. In order to promote health advances through clinical research, government bodies must provide ongoing commitment to support investigator-led research. This support is vital for ensuring that trials conducted by non-industry sponsors that address clinically important questions (such as the IDEA collaboration) can be strengthened in the future. However, substantial government support can be especially difficult to maintain in low-middle income countries with limited financial resources and other competing interests (although this challenge is not unique to low-middle income countries). The critical importance of academic clinical trials should be acknowledged and promoted, which will hopefully also lead to increased funding from foundations and private sources. Collaborations between academic cooperative groups and pharmaceutical industry partners often raise concerns of excessive industry influence on clinical trial design, conduct and reporting. However, with appropriate safeguards for ensuring academic research independence, cooperative-group–industry collaborations can benefit both parties—access to novel agents and supplemental financial support for academic investigator trials, and access to high-quality clinical trial sites and expertise for industry partners.^52^

Box 2. Cancer Breakthroughs 2020

- In 2016 the US government announced Cancer Breakthroughs 2020, formerly known as Cancer Moonshot 2020, comprising a coalition of researchers, oncologists, healthcare funders and pharmaceutical companies.
- Cancer Breakthroughs 2020 aims to accelerate the development of cancer therapies, with a focus on immunotherapy, as well as research into prevention and early detection of cancer.
- Some examples of Cancer Breakthroughs 2020 initiatives include:
  - The National Cancer Institute’s Genomic Data Commons (GDC), a unified system that promotes sharing of genomic and clinical data between researchers.^54^
  - National Cancer Institute (NCI) Formulary, a public-private partnership between the NCI and pharmaceutical and biotechnology companies that provides investigators rapid access to agents for cancer clinical trial use or pre-clinical research.^55^
  - The Partnership for Accelerating Cancer Therapies (PACT), a public-private research collaboration between the National Institute of Health to identify, develop and validate biomarkers for immunotherapy.^56^

## Conclusion

International collaboration in clinical trials has already yielded enormous rewards in oncology, such as the MOSAIC trial that demonstrated the survival benefit associated with the addition of oxaliplatin to adjuvant chemotherapy in stage III colorectal cancer, and the more recent IDEA collaboration investigating the optimal duration of adjuvant therapy in this setting.^23,53^ However, there remain complex barriers to efficient and effective collaboration that require urgent attention. Addressing these challenges requires coordinated worldwide efforts between different stakeholders, including government and regulatory bodies, academia and industry (Box 3). It is only through critical evaluation of past experiences and ongoing efforts toward improvement that the full potential of international trial collaboration can be realised. Understanding the complex history of clinical trials is important in appreciating the resultant intensification of requirements from regulatory bodies. However, these agencies may be actually doing patients and their communities a disservice by inadvertently hindering the clinical trial efforts by increasing the administrative burden, time and resources required to initiate and conduct research—an outcome they undoubtedly neither wished nor intended. Surely it is time for researchers, regulators and those responsible for health systems to show leadership in the global fight against cancer and heed this desperate call to action!

Box 3. Summary of recommendations

- Promotion of the importance of investigator-initiated clinical trials and translational research in improving the care of patients with cancer.
- Regulatory requirements
  - Regulatory reform to streamline and harmonise regulatory, legal and administrative requirements to facilitate more pragmatic, efficient and achievable standards in clinical trial design, conduct and oversight.
  - Development of risk assessment tools and risk-adapted monitoring procedures for quality management in clinical trials.
- Government funding
  - Ongoing commitment from government bodies to fund investigator-led research.
- Logistics
  - Adoption of evidence-based recommendations to improve efficiency and quality in clinical trials, such as promoted by the Clinical Trials Transformation Initiative (CTTI) and the Working Group to Facilitate International Cooperation in Non-Commercial Clinical Trials.
  - Promotion of routine data-sharing practices and systems within the cancer research community to prevent unnecessary duplication of trials and maximise the potential of existing oncology clinical trial data.
  - Greater use of centralised institutional review boards to streamline ethical approval processes.

## Data Availability

Not applicable

## References

[1] Pocock S, Calvo G, Marrugat J, Prasad K, Tavazzi L, Wallentin L (2013). International differences in treatment effect: do they really exist and why?. Eur. Heart J..

[2] Simes RJ, O’connell RL, Aylward PE, Varshavsky S, Diaz R, Wilcox RG (2010). Unexplained international differences in clinical outcomes after acute myocardial infarction and fibrinolytic therapy: lessons from the Hirulog and Early Reperfusion or Occlusion (HERO)-2 trial. Am. Heart J..

[3] Bassand J-P, Martin J, Rydén L, Simoons M (2002). The need for resources for clinical research: the European Society of Cardiology calls for European, international collaboration. Lancet.

[4] Minisman G, Bhanushali M, Conwit R, Wolfe GI, Aban I, Kaminski HJ (2012). Implementing clinical trials on an international platform: challenges and perspectives. J. Neurol. Sci..

[5] Søreide K, Alderson D, Bergenfelz A, Beynon J, Connor S, Deckelbaum DL (2013). Strategies to improve clinical research in surgery through international collaboration. Lancet.

[6] Organisation for Economic Co-operation and Development Global Science Forum. Facilitating International Cooperation in Non-Commercial Clinical Trials. OECD; October 2011. https://www.oecd.org/sti/sci-tech/49344626.pdf (2018).

[7] Wileman H, Mishra A (2010). Drug lag and key regulatory barriers in the emerging markets. Perspect. Clin. Res..

[8] Directive 2001/20/EC of the European Parliament and the Council of 4 Apr 2001 on the approximation of laws, regulations and administrative provisions of the Member States relating to the implementation of good clinical practice in the conduct of clinical trials on medicinal products for human use. *Official J. Eur. Community* **L121**, 34–44 (2001). 16276663

[9] Hemminki Akseli, Kellokumpu-Lehtinen Pirkko-Liisa (2006). Harmful impact of EU clinical trials directive. BMJ.

[10] Hartmann M (2012). Impact assessment of the European Clinical Trials Directive: a longitudinal, prospective, observational study analyzing patterns and trends in clinical drug trial applications submitted since 2001 to regulatory agencies in six EU countries. Trials (J. Artic.).

[11] Regulation (EU) No 536/2014 of the European Parliament and of the Council of 16 April 2014 on clinical trials on medicinal products for human use, and repealing Directive 2001/20/EC. *Official J. Eur. Union* **L158**, 1–76 (2014).

[12] Weigmann K (2015). The ethics of global clinical trials: in developing countries, participation in clinical trials is sometimes the only way to access medical treatment. What should be done to avoid exploitation of disadvantaged populations?. EMBO Rep..

[13] Regulation (EU) 2016/679 of the European Parliament and of the Council of 27 April 2016 on the protection of natural persons with regard to the processing of personal data and on the free movement of such data, and repealing Directive 95/46/EC (General Data Protection Regulation). *Official J. Eur. Union* **L119**, 1 (2016).

[14] Clarke N., Vale G., Reeves E. P., Kirwan M., Smith D., Farrell M. et al. GDPR: an impediment to research? *Ir. J. Med. Sci.*10.1007/s11845-019-01980-2 (2019). 10.1007/s11845-019-01980-2 30734900

[15] Metzger-Filho O, De Azambuja E, Bradbury I, Saini KS, Bines J, Simon SD (2013). Analysis of regional timelines to set up a global phase III clinical trial in breast cancer: the adjuvant lapatinib and/or trastuzumab treatment optimization experience. Oncologist.

[16] Hemminki E (2015). Actors involved in the regulation of clinical research: comparison of Finland to England, Canada, and the USA. Health Res. Policy Syst. (J. Artic.).

[17] Majumdar SR, Roe MT, Peterson ED, Chen AY, Gibler WB, Armstrong PW (2008). Better outcomes for patients treated at hospitals that participate in clinical trials. Arch. Intern. Med..

[18] Ozdemir BA, Karthikesalingam A, Sinha S, Poloniecki JD, Hinchliffe RJ, Thompson MM (2015). Research activity and the association with mortality. PLoS ONE.

[19] Downing A., Morris E. J., Corrigan N., Sebag-Montefiore D., Finan P. J., Thomas J. D. et al. High hospital research participation and improved colorectal cancer survival outcomes: a population-based study. *Gut* gutjnl-2015-311308 (2016). 10.1136/gutjnl-2015-311308 PMC5256392 27797935

[20] Zalcberg JR, Friedlander M (2018). The value of participating in clinical trials: the whole is greater than the sum of its parts. Med. J. Aust..

[21] Atal I, Trinquart L, Porcher R, Ravaud P (2015). Differential globalization of industry- and non-industry–sponsored clinical trials. PLoS ONE.

[22] Seruga B, Sadikov A, Cazap EL, Delgado LB, Digumarti R, Leighl NB (2014). Barriers and challenges to global clinical cancer research. Oncologist.

[23] Grothey A, Sobrero AF, Shields AF, Yoshino T, Paul J, Taieb J (2018). Duration of adjuvant chemotherapy for stage III colon cancer. N. Engl. J. Med..

[24] Rogozinska E, Marlin N, Thangaratinam S, Khan KS, Zamora J (2017). Meta-analysis using individual participant data from randomised trials: opportunities and limitations created by access to raw data. Evid. Based Med..

[25] Taichman DB, Sahni P, Pinborg A, Peiperl L, Laine C, James A (2017). Data sharing statements for clinical trials: a requirement of the International Committee of Medical Journal Editors. Ann. Intern. Med..

[26] Savage CJ, Vickers AJ (2009). Empirical study of data sharing by authors publishing in PLoS journals. PloS ONE.

[27] Reidpath DD, Allotey PA (2001). Data sharing in medical research: an empirical investigation. Bioethics.

[28] Project Data Sphere. https://www.projectdatasphere.org/ (2018).

[29] Emdin C. A., Odutayo A., Hsiao A. J., Shakir M., Hopewell S., Rahimi K., Altman D. G. (2015). Association between randomised trial evidence and global burden of disease: cross sectional study (Epidemiological Study of Randomized Trials--ESORT). BMJ.

[30] Li R., Barnes M., Aldinger C., Bierer B. Global clinical trials: ethics, harmonization and commitments to transparency. *Glob. Health* **4**, 2 http://harvardpublichealthreview.org/wp-content/uploads/2015/04/HPHRv5-Li-Barnes-Aldinger-Bierer-Global-Clinical-Trials.pdf (2015).

[31] Glickman SW, McHutchison JG, Peterson ED, Cairns CB, Harrington RA, Califf RM (2009). Ethical and scientific implications of the globalization of clinical research. N. Engl. J. Med..

[32] Hyder AA, Wali S, Khan A, Teoh N, Kass N, Dawson L (2004). Ethical review of health research: a perspective from developing country researchers. J. Med. Ethics.

[33] Thiers FA, Sinskey AJ, Berndt ER (2008). Trends in the globalization of clinical trials. Nat. Rev. Drug Discov..

[34] Council for International Organizations of Medical Sciences. International ethical guidelines for biomedical research involving human subjects. 4th edn. (Geneva, 2016). https://cioms.ch/wp-content/uploads/2017/01/WEB-CIOMS-EthicalGuidelines.pdf. 14983848

[35] Vickers AJ (2014). Clinical trials in crisis: four simple methodologic fixes. Clin. Trials.

[36] Tenaerts P, Madre L, Landray M (2018). A decade of the Clinical Trials Transformation Initiative: what have we accomplished? What have we learned?. Clin. Trials.

[37] Yusuf S, Bosch J, Devereaus PJ, Collins R, Baigent C, Granger C (2008). Sensible guidelines for the conduct of large randomized trials. Clin. Trials.

[38] Clinical Trials Transformation Initiative. http://www.ctti-clinicaltrials.org (2019).

[39] Lang T, Siribaddana S (2012). Clinical trials have gone global: is this a good thing?. PLoS Med..

[40] Valdivieso M, Corn BW, Dancey JE, Wickerham DL, Horvath LE, Perez EA (2015). The globalization of cooperative groups. Semin. Oncol..

[41] McMahon AD, Conway DI, MacDonald TM, McInnes GT (2009). The unintended consequences of clinical trials regulations. PLoS Med..

[42] International Council for Harmonisation of Technical Requirements for Pharmaceuticals for Human Use. Integrated Addendum to ICH E6(E1): Guidelines for good clinical practice E6(R2). (International Council for Harmonisation of Technical Requirements for Pharmaceuticals for Human Use, Geneva, 2016).

[43] Tudur Smith C, Stocken DD, Dunn J, Cox T, Ghaneh P, Cunningham D (2012). The value of source data verification in a cancer clinical trial. PLoS ONE.

[44] Dittrich C, Negrouk A, Casali PG (2015). An ESMO-EORTC position paper on the EU clinical trials regulation and EMA’s transparency policy: making European research more competitive again. Ann. Oncol..

[45] Union for International Cancer Control. TNM Classification of Malignant Tumours. 8th ed. (Wiley-Blackwell, 2016).

[46] World Health Organization. WHO International Classification of Diseases. http://www.who.int/classifications/icd/en (2018).

[47] National Cancer Institute. Common Toxicity Criteria for Adverse Events. https://ctep.cancer.gov/protocolDevelopment/electronic_applications/ctc.htm (2018).

[48] Eisenhauer E, Therasse P, Bogaerts J, Schwartz L, Sargent D, Ford R (2009). New response evaluation criteria in solid tumours: revised RECIST guideline (version 1.1). Eur. J. Cancer.

[49] Seymour L, Bogaerts J, Perrone A, Ford R, Schwartz LH, Mandrekar S (2017). iRECIST: guidelines for response criteria for use in trials testing immunotherapeutics. Lancet Oncol..

[50] Trimble EL, Abrams JS, Meyer RM, Calvo F, Cazap E, Deye J (2009). Improving cancer outcomes through international collaboration in academic cancer treatment trials. J. Clin. Oncol..

[51] CEO Roundtable on Cancer. http://www.ceoroundtableoncancer.org (2018).

[52] Bressler LR, Schilsky RL (2008). Collaboration between cooperative groups and industry. J. Oncol. Pract..

[53] André T, Boni C, Mounedji-Boudiaf L, Navarro M, Tabernero J, Hickish T (2004). Oxaliplatin, fluorouracil, and leucovorin as adjuvant treatment for colon cancer. N. Engl. J. Med..

[54] National Institutes of Health. Newly launched Genomic Data Commons to facilitate data and clinical information sharing. https://www.nih.gov/news-events/news-releases/newly-launched-genomic-data-commons-facilitate-data-clinical-information-sharing (2019).

[55] National Cancer Institute. NCI Formulary. https://nciformulary.cancer.gov/ (2019).

[56] Foundation for the National Institutes of Health. Partnership for Accelerating Cancer Therapies (PACT). https://fnih.org/what-we-do/programs/partnership-for-accelerating-cancer-therapies (2019).

